# Application of Extrusion-Based Hydrogel Bioprinting for Cartilage Tissue Engineering

**DOI:** 10.3390/ijms18071597

**Published:** 2017-07-23

**Authors:** Fu You, B. Frank Eames, Xiongbiao Chen

**Affiliations:** 1 Division of Biomedical Engineering, College of Engineering, University of Saskatchewan, 57 Campus Drive, Saskatoon, SK S7N 5A9, Canada; fuy618@campus.usask.ca (F.Y.); b.frank@usask.ca (B.F.E.); 2 Department of Anatomy and Cell Biology, College of Medicine, University of Saskatchewan, 107 Wiggins Road, Saskatoon, SK S7N 5E5, Canada; 3 Department of Mechanical Engineering, College of Engineering, University of Saskatchewan, 57 Campus Drive, Saskatoon, SK S7N 5A9, Canada

**Keywords:** cartilage tissue engineering, extrusion-based bioprinting, hydrogels, bio-inks, self-supporting hydrogel bioprinting, hybrid bioprinting

## Abstract

Extrusion-based bioprinting (EBB) is a rapidly developing technique that has made substantial progress in the fabrication of constructs for cartilage tissue engineering (CTE) over the past decade. With this technique, cell-laden hydrogels or bio-inks have been extruded onto printing stages, layer-by-layer, to form three-dimensional (3D) constructs with varying sizes, shapes, and resolutions. This paper reviews the cell sources and hydrogels that can be used for bio-ink formulations in CTE application. Additionally, this paper discusses the important properties of bio-inks to be applied in the EBB technique, including biocompatibility, printability, as well as mechanical properties. The printability of a bio-ink is associated with the formation of first layer, ink rheological properties, and crosslinking mechanisms. Further, this paper discusses two bioprinting approaches to build up cartilage constructs, i.e., self-supporting hydrogel bioprinting and hybrid bioprinting, along with their applications in fabricating chondral, osteochondral, and zonally organized cartilage regenerative constructs. Lastly, current limitations and future opportunities of EBB in printing cartilage regenerative constructs are reviewed.

## 1. Bioprinting Is a Promising Technique to Process Hydrogel for Fabricating Cartilage Constructs

Bioprinting of personalized complex tissue grafts is promising for overcoming the current challenges of cartilage tissue engineering (CTE). Cartilage is a highly hydrated and specialized tissue to provide a low-friction, wear-resistant, and load-bearing surface in diarthrodial joints for efficient joint movement [1]. Unfortunately, the structure and function of the cartilage are frequently disrupted or lost with trauma or aging; moreover, there is no sufficient heal response for regeneration as cartilage shows little self-repair tendency. These defects or injuries last for years and eventually lead to arthritis [2]. To address this problem, tissue engineering (TE) approaches aiming to engineer constructs to regenerate cartilage defects are under active investigation. Ideally, the tissue-engineered constructs for CTE should fill cartilage defects, resemble extracellular matrix (ECM), hold cells in place, and retain a space for the growing tissue [3,4]. To this end, hydrogel has been illustrated promising due to the fact that it closely mimics native ECM and thus providing a 3D culture microenvironment favorable for encapsulated cells to retain the rounded morphology and chondrogenic phenotype [5,6,7]. Furthermore, hydrogels allow for achieving high cell seeding density and homogenous cell distribution throughout scaffold [6,8,9,10,11,12,13,14], and transmitting external stimuli to embedded cells so as to direct growth and formation of the regenerating cartilage [15,16]. Several disadvantages of hydrogels, however, have also been identified, such as weak mechanical strength and stability. It is also hard to handle and process hydrogels into cartilage regenerative constructs with desired internal structure and external shape. To overcome these problems, the bioprinting technique has been rapidly developing and gaining interest for fabrication of customized cartilage constructs.

Although some reviews on bioprinting of tissues and organs are available, investigation into the extrusion-based bioprinting (EBB) of cartilage constructs from bio-inks has not been well-documented. This article presents a brief review of the application of EBB for fabricating cartilage constructs from bio-inks, covering its working principles, applicable cell sources and materials, printability, printed cartilage constructs, as well as future perspectives of bioprinting cartilage.

## 2. Extrusion-Based Bioprinting and Bio-Inks for Cartilage Tissue Engineering

### 2.1. Extrusion-Based Bioprinting

Rapid prototyping (RP), also known as solid freeform fabrication, refer to a series of techniques that manufacture objects through sequential delivery of energy and/or material in a layer-by-layer manner per computer aided design (CAD) data. The external shape and internal architecture of the scaffold can be defined by either 3D computer models or clinical imaging data (e.g., the defect area of the patient can be scanned by magnetic resonance imaging or computed tomography) [17,18]. Once the external/internal geometric information is determined, the RP system is programed to fabricate the scaffold as designed.

**Figure 1:**
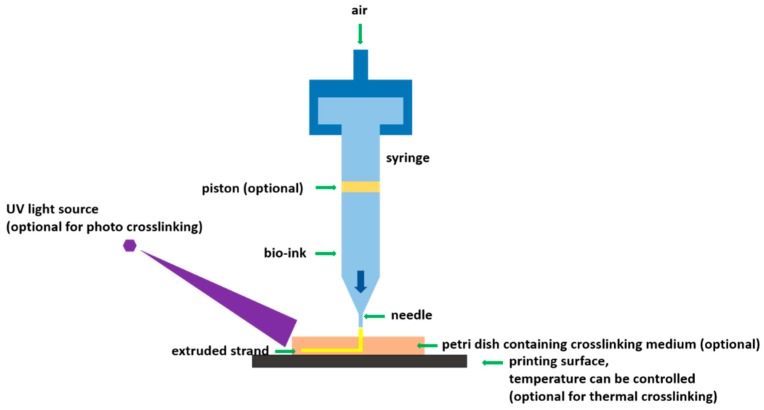
Schematic of extrusion-based bioprinting using various crosslinking mechanisms.

Among various RP techniques, EBB stands out for its unique advantages. It allows for production of 3D tissue constructs from bio-inks by a layer-by-layer deposition process in a designed way [19]. EBB also allows for higher cell seeding density, higher printing speed to facilitate scalability, and relatively less process-induced cell damage compared to other techniques [20]. EBB can print continuous cylindrical filaments from almost all types of bio-inks to high cell density aggregates of a wide range of viscosities. Once the bio-ink is printed, it can be crosslinked by ionic, photo, and/or thermal crosslinking mechanisms (Figure 1). Given the complexity of biological tissue, multiple bio-inks are often used to fabricate a tissue construct, which is also achievable by using EBB with multiple printing heads.

### 2.2. Bio-Inks

Hydrogel precursors and living cells are two important components of bio-ink formulations. Cell sources and hydrogel types employed for encapsulating chondrogenic cells are reviewed below.

#### 2.2.1. Applicable Cell Sources

The choice of cells is a central problem to any modality of TE. For cartilage bioprinting, several factors need to be taken into consideration when choosing suitable cell sources: (i) cells must be robust enough to survive any shear stress and pressure during the printing process; (ii) cells must proliferate well; (iii) cells must possess biosynthesis levels (e.g., of proteoglycans, Collagen type II) comparable with native chondrocytes so they can maintain their biological functions [21]. So far, the use of chondrocytes over stem cells for cartilage bioprinting is predominant (Table 1).

Consistent with the distinct zonal structure of native articular cartilage [22], chondrocytes from different zones show different characteristics of biosynthesis levels. Superficial zone has a dense network of collagen fibers that are parallel to the articular surface, while collagen fibers are randomly arranged in the middle region and perpendicular to the subchondral bone in the deep zone [23]. The content of the other important component in cartilage, proteoglycan, is lowest in the superficial zone and increases through the middle and deep zones [24]. Limited number of chondrocytes in articular cartilage makes it necessary to expand chondrocytes before use. The monolayer expansion process usually leads to chondrocyte dedifferentiation with decreased GAG synthesis and Collagen type II expression [25,26]. Most studies typically use chondrocyte mixtures from full-thickness cartilage [27,28,29] to obtain higher cell populations. Recently, more attention has been focused on employing zonal chondrocytes to achieve different purposes. For example, deep zone chondrocytes are utilized to engineer a functional osteochondral interface by coculturing with calcium phosphate [30]. Chondrocytes isolated from the superficial layer exhibit increased proteoglycan 4 expression, and thus superficial chondrocytes are promising to be used as the cell source for engineering articular surface [1]. Articular chondrocytes provide researchers with a unique opportunity to replicate the native zonal structure by embedding and culturing zonal chondrocytes in different layers of gels, although it is still elusive if this is a promising approach or an overcomplicated strategy [31]. Donor site morbidity during harvesting of joint cartilage further limits the use of articular chondrocytes [32]. Therefore, nasoseptal chondrocytes, as another autologous chondrocyte source, is also explored for bioprinting cartilage constructs [33,34]. Another promising cell type is the multipotent mesenchymal stem cell (MSC), which can be derived from multiple tissues, such as bone marrow, adipose tissues, synovium, periosteum, and muscle. These stem cells can be differentiated to undergo chondrogenesis with the supplement of specific growth factors [35,36], such as transforming growth factor beta family [37] and therefore they have been explored to be used in CTE [38,39,40,41,42,43,44].

#### 2.2.2. Applicable Hydrogel-Forming Polymers for Formulating Bio-Inks

Hydrogel cross-linking mechanisms are generally categorized into “physical” crosslinking and “chemical” crosslinking. Physical (thermal [69,70] ionic [71] and photo [72]) crosslinking include reversible entangled chains, hydrogen bonding, etc. while chemical (enzyme [73] and pH [74]) crosslinking are permanent junctions formed by irreversible, covalent bonds. Hydrogel can be classified into two groups based on their sources: natural hydrogels (e.g., agarose, alginate, cellulose, gelatin, gellan gum, hyaluronic acid, collagen, fibrin) and synthetic hydrogels (e.g., Pluronic^®^ F127, PEG, and PVA). Hydrogels that are biocompatible for encapsulating stem cells or chondrogenic cells for CTE are summarized and reviewed (Table 2). There are pros and cons to each type of these hydrogels and researchers attempted to modify these polymers to improve their properties like bioactivity, mechanical properties, and printability.

## 3. Important Properties of Bio-Inks

### 3.1. Biocompatibility

Biocompatibility must be considered before the application of any material for TE and regenerative medicine. Biocompatibility refers to the ability of a biomaterial to perform its desired function without eliciting any undesirable biological effects [122]. For the purposes of this review, a bioprinted hydrogel must be cytocompatible and nonimmunogenic, and have nontoxic byproducts of degradation without eliciting any detrimental effects from the time of bioprinting to in vitro maturation and in vivo implantation [123]. The main factor that could influence the biocompatibility given the same material lies in the bioprinting process, which means the whole printing process needs to be cytocompatible. In most cases, bio-inks are stored as liquids in a reservoir prior to being dispensed onto the printing surface and a crosslinking process is followed to solidify the bio-inks. The cytocompatibility of this process is characterized by the cell viability test using live/dead staining [124]. To elevate the cell viability, bio-inks are designed to minimize the stress-induced damage to cells due to the sensitivity of cells encapsulated in the bio-inks. In the cases of printing mechanisms involving the use of heating or pressure, the heating temperatures are kept within the range favoring cell survival and the pressure is maintained as low as possible.

### 3.2. Printability

Printability of a bio-ink, once printed in a layer-by-layer fashion, is its ability to form and maintain a structure as designed with structural fidelity and integrity. Printability is considered to be associated with surface tension, viscosity, rheological properties, and crosslinking mechanisms. Standardized tests to quantify the printability still do not exist, and an optical examination method is usually adopted to do a geometry comparison (e.g., pore size, fiber diameter) between generated constructs and CAD data [125,126].

#### 3.2.1. First-Layer Formation

The printing and formation of the first layer of bio-inks play an important role for fabricating the whole construct. A relatively large contact angle between dispensed bio-inks and the substrate help to maintain the vertical dimension of printed bio-inks and avoid the flattening of the printed hydrogel precursor solution. The interaction between printed bio-inks and substrate is crucial, since suitable interaction helps to anchor the whole bioprinted construct on the printing surface and avoids possible deformation and undesired movement during the layer-by-layer bio-inks deposition process. Unfortunately, most receiving surfaces such as glass or plastic have poor contact angles with bio-inks and it is difficult to establish any interaction between receiving surface and dispensed bio-inks. These issues could be addressed by either printing hydrogels in a hydrophobic high-density fluid, such as perfluorotributylamine [78], or coating a thin layer of chemicals, such as 3-(trimethoxysilyl) propyl methacrylate, on the printing surface [127] to enhance their hydrophobicity. Polyethylenimine was used successfully in our group to pre-treat the culture plates to establish an electrostatic interaction between printed cell-laden hydrogel and the receiving surface [128].

#### 3.2.2. Viscosity

Viscosity describes the internal resistance of a fluid to flow upon application of stress. The viscosity of a polymer solution is determined by its concentration, molecular weight, and temperature. Higher polymer concentration and molecular weight are associated with higher viscosity. Typically, sufficient viscosity of bio-inks leads to good printability, since it can help the bio-inks to overcome the surface-tension-driven droplet formation and be drawn to form continuous strands. Sufficient viscosity will also help the dispensed strands to maintain the cylinder shape and keep adjacent strands from merging together, which also explains why thermoplastic polymers are usually printed with higher accuracy and resolution than hydrogels. However, cells thrive best in an aqueous environment, in which their matrix deposition is not limited by the dense polymer network [129]. Bio-inks with high viscosity require high pressure to expel them out of the dispensing needle; in this case, the embedded cells are exposed to a high shear force, which may impair cell viability [130].

The viscosity of a bio-ink solution is mainly determined by the polymer concentration and molecular weight. Given that bio-inks with high concentrations may not be favorable for cell proliferation/migration and ECM formation [129], it is reasonable to choose low concentrations of high molecular weight polymers for better printability in bioprinting. This also explains the success of natural polymers in the bioprinting area.

#### 3.2.3. Shear Thinning

Shear thinning is another desirable feature for bio-inks that will help to improve the printability, and it refers to the fact that viscosity decreases as shear rate increases [131]. Polymer solutions with higher concentrations show more obvious shear thinning. When bio-inks are exposed to high shear rates inside a nozzle during bioprinting, a decreased viscosity or shear stress will be present, which favors the survival of embedded cells. Meanwhile, a sudden decrease of shear rates upon deposition causes a sharp increase in viscosity, resulting in a high printing fidelity.

#### 3.2.4. Crosslinking Mechanisms

The printability is also influenced by how easily and efficiently materials can be crosslinked. EBB usually requires printing a cell-laden polymer solution followed by initiating gelation immediately after extrusion. The cell-laden polymer solution must be either prepared quite viscous or crosslinked rapidly after dispensing onto the printing surface to achieve good printability and shape fidelity. However, high viscosity is not ideal for its application in TE and impedes cells survival and proliferation [132,133]. Therefore, a relatively rapid crosslinking process is usually desirable in the printing process. Currently, ionic, photo, and thermal crosslinking are most commonly used crosslinking mechanisms in bioprinting (Table 2).

### 3.3. Strategies to Strengthen Mechanical Properties of Engineered Cartilage Construct

Engineered cartilage should maintain sufficient mechanical properties after bioprinting to provide embedded cells with a stable environment for attachment, proliferation, and differentiation. Particularly for cartilage bioprinting in CTE, mechanical properties are crucial because the functions of cartilage mainly rely on their mechanical performance. Mechanical properties of hydrogel are intrinsically weak compared to cartilage [134]. Strategies have been developed to strengthen the initial mechanical performance of engineered constructs.

Research has supplemented hydrogel with mineral particles (e.g., hydroxyapatite) to create composite hydrogels, by combining organic and inorganic phases to obtain desirable properties including the improvement of mechanical properties and enhancement of biological properties [135,136]. In CTE, the presence of calcium phosphate has been shown to promote chondrocyte hypertrophy and Collagen type X deposition and thus improve the regeneration of calcified cartilage [30,137]. Moreover, hydroxyapatite would be a good supplement in scaffolding materials in CTE to recruit endogenous cells in vivo to regenerate articular surface without cell transplantation [138].

A novel approach reinforced hydrogel constructs by incorporating printed polycaprolactone (PCL) scaffolds. Hydrogel precursors were poured and perfused into the printed porous PCL scaffold and crosslinked. In this way, the stiffness of the resulting constructs could be tailored to that of native cartilage by reinforcement with high-porosity PCL scaffolds [139]. Fabricating cartilage constructs by alternating printing injected-printed hydrogels and electrospun thermoplastic polymer fibers is also feasible [108]. It would be a promising technique if electrospun thermoplastic polymer fibers can be incorporated into EBB to print constructs with native mechanical characteristics.

A higher mechanical strength can also be achieved by blending multiple polymers and varying the molar ratio of bio-ink components. From instance, nanocellulose and alginate composite bio-ink was synthesized and printed to fabricate chondrocyte-laden constructs. Increasing the alginate fraction in bio-ink formula would lead to an increase in compressive modulus of printed constructs [34].

Making use of the crosslinking mechanism is also an efficient way to enhance the mechanical properties of the printed constructs. For example, a three-step method was used to crosslink alginate hydrogel for improved elastic stiffness; furthermore, the three steps are the primary calcium ionic cross-linking to increase the initial viscosity of alginate, secondary calcium ionic crosslinking to solidify the printed structure, and tertiary barium ionic crosslinking to strengthen elastic stiffness [140].

Another effective way to enhance the mechanical properties is the use of hybrid bioprinting to co-deposit hydrogels and thermoplastic polymers alternately. Cell-laden hydrogels are supported by printed thermoplastic polymers; thus, these hybrid constructs possess mechanical characteristics that are mainly provided by the printed thermoplastic polymer frame, which is significantly higher than the hydrogel-only constructs [141]. Meanwhile, by designing and changing the architecture of the thermoplastic polymer framework parameters, including molecular weight of polymer, strand size, strand spacing, and strand orientation, the mechanical properties of the construct can be tuned [142]. A covalent bonding based on methacrylate groups between thermoplastic polymer methacrylated poly(hydroxymethylglycolide-co-e-caprolactone)/PCL (pHMGCL/PCL) and gelatin methacrylamide (GelMA) hydrogel can also be established to improve binding in the interface of two materials and further elevate the mechanical performance of the engineered construct [29].

If a scaffold is designed to initially promote engineered tissue formation in vitro prior to implantation in vivo, then they are not required to exactly match the mechanical properties of natural cartilage at the initial stage. Thereby, many hydrogel-based cartilage bioprinting research still focus on formulating bio-inks to favor the synthesis of cartilaginous ECM instead of their initial mechanical strength with the hope that the ECM generated by the cells in vitro provides sufficient mechanical properties upon implantation in vivo.

## 4. Cartilage Constructs Bioprinting Approaches

Current cartilage constructs are mainly printed based on two approaches: (i) direct printing of cartilage constructs from bio-inks (called the self-supporting hydrogel bioprinting) and (ii) alternating printing of bio-inks and thermoplastic-polymer network (called the hybrid bioprinting). The advantages of self-supporting hydrogel bioprinting rests on their mild and physiological crosslinking conditions and its relatively simple process as compared to hybrid bioprinting. However, the self-supporting bioprinting requires a high level of printability of bio-inks and the printed hydrogel constructs typically have week mechanical properties [128]. In contrast, the thermoplastics network printed in hybrid bioprinting can offer a sufficient mechanical support to the subsequently dispensed hydrogel strands for being crosslinked. Therefore, hybrid bioprinting can print a broader range of bio-inks than self-supporting hydrogel bioprinting. Nevertheless, the high temperature for melting thermoplastic polymers in hybrid bioprinting may impair cell viability. Additionally, hybrid bioprintng may introduce extra printing errors due to its complex process and heating-related stresses within printed constructs [143].

### 4.1. Self-Supporting Hydrogel Bioprinting

Self-supporting hydrogel bioprinting approaches form cartilage constructs for CTE application by printing stem cell- or chondrocyte-laden natural and synthetic hydrogels [144]. Chondrocytes and stem cells embedded within alginate hydrogels has been demonstrated to be viable and metabolically active [145]. Rapid crosslinking makes alginate a commonly used component in bio-inks to print cartilage constructs. A highly printable bio-ink consisted of alginate and nanocellulose was formulated. The printed constructs supported the culture of human nasoseptal chondrocytes and had the potential to be printed into more complex shapes [34]. Alginate has also been sulfated to bind growth factors such as fibroblast growth factor (FGF), transforming growth factor (TGF) without losing its printability [146,147]. A chondrocyte-laden construct consisting of sulfated alginate and nanocellulose still provided good printability and Collagen type II deposition [148,149]. Lack of sufficient cell adhesion sites still limits the application of alginate in CTE. By incorporating BioCartilage (cartilage extracellular matrix particles) and gellan in alginate, the bioactivity and printability of the bio-ink was significantly improved and the resulting patient-specific cartilage grafts showed good mechanical property and biological properties [27].

Hyaluronic acid (HA), as an essential component of cartilage ECM, can mediate cellular signaling, wound repair, and ECM organization due to its structural and biological properties [150]. More recently, HA is increasingly explored as a “building block” in various bio-inks formulations for cartilage bioprinting in CTE because of its viscoelastic and bioactive properties [151]. Nevertheless, one major drawback of unmodified HA for cartilage bioprinting is the poor stability owing to its water solubility. To address the problem of the poor stability of printed HA, the photo-crosslinkable dextran derivate or acrylated Pluronic was added to improve mechanical properties and the printability of the material. Moreover, embedded chondrocytes demonstrated good compatibility with this bio-inks formulation [152,153].

Although gelatin gel has been shown to support chondrocyte viability and differentiation, its low viscosity and de-crosslinking at 37 °C make it hard to print [154]. Therefore, gelatin is usually modified to become photo-crosslinkable by a straightforward reaction with an acrylate or methacrylate agent [72,155]. For example, a study [102] explored the functionalization, preparation and use of cell-laden gelatin methacryloyl (GelMA)-based hydrogels as modular tissue culture platforms. For improved printability of gelatin, HA was also incorporated in GelMA and printed chondrocyte-laden constructs supported the viability of embedded chondrocytes and cartilaginous tissue formation [50].

Acrylation is also commonly used with synthetic hydrogels to facilitate cartilage bioprinting. An example is printing poly (ethylene glycol) dimethacrylate (PEGDMA) together with human chondrocytes to repair defects with osteochondral plugs through a layer-by-layer manner. The printed construct showed a higher mechanical property of 395.73 kPa than most printed natural hydrogels. This study demonstrated that hydrogel bioprinting is a feasible approach of producing cartilage constructs with anatomic characteristics to accurate targeted locations. The embedded human chondrocyte viability was 89% and showed an elevated glycosaminoglycan (GAG) content. Additionally, printed cartilage constructs firmly attached to the surrounding tissue and showed even greater proteoglycan deposition at the interface of implant and native cartilage [48].

Improving the integrity between the engineered cartilage and subchondral bone remains a challenge. In this regard, a self-supporting hydrogel construct was printed onto the printed bone paste (consisting of demineralized bone matrix and powdered gelatin) to mimic the cartilage and subchondral bone respectively [156]. Heterogeneous cell-laden high-viscosity alginate hydrogel constructs were printed with distinct parts for human chondrocytes and osteogenic progenitors for potential use as osteochondral grafts. Embedded cells stayed in their compartment of the printed scaffold for the whole culture period and viability remained high throughout the printing and culture process and cartilage and bone ECM formation were observed both in vitro and in vivo [157]. The reported cartilage constructs fabricated by self-supporting hydrogel bioprinting are summarized in Table 3.

To sum up, self-supporting hydrogel bioprinting of cartilage constructs can be processed under cytocompatible conditions and printed constructs are generally shown to support cartilage ECM biosynthesis. Current research emphasis is focused on formulating bio-inks to achieve high printability and improving the mechanical performance of printed constructs. The relatively weak mechanical properties of printed hydrogel-based cartilage constructs limit its application to regenerating focal cartilage defects, where most exerted force is born by its surrounding tissue. To overcome these issues, hybrid cartilage bioprinting by alternating printing of bio-inks and thermoplastic polymers fibers (hybrid bioprinting) has been brought forward.

### 4.2. Hybrid Bioprinting

A hybrid construct combining advantages of hydrogel and thermoplastics has been brought forward, offering potential for application in CTE [141]. Scaffolds made from thermoplastic polymers provide stronger structural properties, and hydrogels provide a biologically favorable, highly hydrated microstructure like native cartilage ECM for chondrocytes. By alternately printing thermoplastic polymer and cell-laden hydrogel, hybrid cartilage construct is yielded. This mechanism makes a broader range of bio-inks types available for use compared to bioprinting of hydrogels alone, since requirements for viscosity and gelling speed are less stringent [141]. Additionally, engineered cartilage fabricated by hybrid bioprinting possesses adequate mechanical characteristics, since the thermoplastic polymer framework mainly provides the mechanical property of the constructs [141].

By applying this state-of-the-art printing technology, human nasoseptal chondrocyte-laden alginate hydrogel with a supportive PCL structure was printed [33]. The study demonstrated in vitro and in vivo applications of hybrid constructs encapsulating chondrocytes and growth factors in CTE. Another trial explored the feasibility to use embryonic chick chondrocytes as cell sources for hybrid printing and comprehensively studied biological performance of the embedded chondrocytes. Cell viability, proliferation, and cartilage ECM biosynthesis were all kept at high levels in hybrid constructs, confirming the validity of the hybrid bioprinting for effective CTE [160]. Given the bioinert nature of alginate, it is not an ideal material for encapsulating chondrocytes and maintaining their functionality. Therefore, a study printed hybrid tissue analogues by dispensing decellularized ECM (dECM) instead of alginate in the abovementioned hybrid bioprinting system. The results showed the versatility and flexibility of hybrid bioprinting process using various tissue-specific dECM bio-inks, including adipose, cartilage and heart tissues, which can provide bioactive cues for embedded cells [143].

Hybrid bioprinting also showed good suitability to fabricate osteochondral constructs, enabling researchers to use different bio-inks in cartilage portion and bone portion. A mechanically stable 3D dual cell-laden construct consisting of osteoblasts and chondrocytes for osteochondral tissue engineering using a multi-head extrusion-based printing system was successfully printed. Two different alginate solutions with encapsulated osteoblasts or chondrocytes were deposited into the previously printed PCL framework [161]. A more recent study from the same research group successfully bioprinted a multilayered construct with three distinct layers by varying the hydrogel materials and incorporated growth factors using a similar hybrid printing process and achieved the regeneration of osteochondral defects in the knee joints of rabbits [162]. Overviews of hybrid bioprinting for fabricating osteo (chondral) constructs reviewed in Table 4.

These studies show the promise of hybrid bioprinting as an advanced fabrication technique for CTE. However, mechanical stimuli exerted on hybrid construct would probably be mainly withstood by the polymeric scaffolds instead of chondrocyte-laden hydrogel because of stress shielding [163]. This might be an issue when considering mechanical stimuli can positively mediate chondrocytes biosynthetic behavior [164] and cartilage tissue remodeling [165]. Therefore, further studies need to be carried out to determine the influence of mechanical stimuli on the engineered hybrid constructs.

## 5. Zonal Cartilage Bioprinting

**Figure 2:**
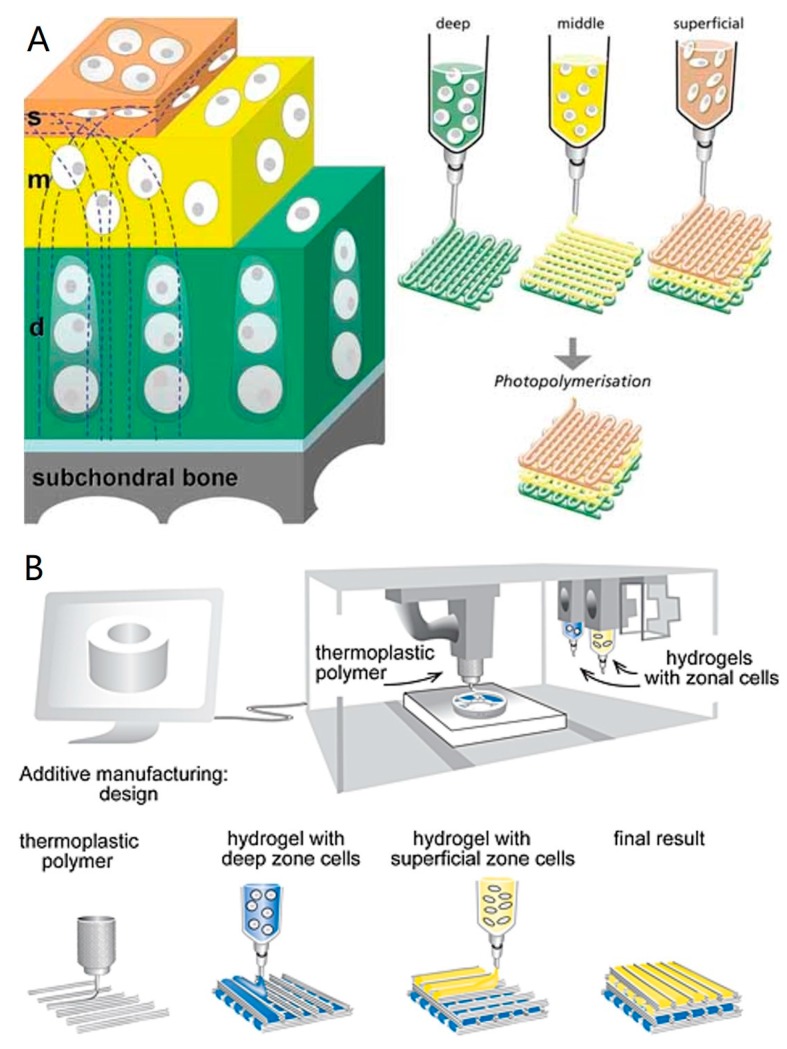
(**A**) Schematic of self-supporting hydrogel bioprinting for fabrication of zonal cartilage constructs. Zonal constructs are printed with chondrocytes from the superficial, middle, and deep zones incorporated in distinct hydrogel precursors in defined geometries. Reproduced with permission. Copy right 2009, Wiley Online Library [144]; (**B**) Schematic of hybrid bioprinting for fabrication of zonal cartilage constructs. Alternating steps of printing polymer and zonal cell-laden hydrogels are performed to obtain zonal constructs Reproduced with permission. Copyright 2015, Wiley Online Library [31].

Zonal cartilage constructs that reflect the native structural depth-dependent characteristics of articular cartilage could have advantages over homogeneous constructs. A zonal cartilage construct can be achieved by the following strategies: (1) using zonal chondrocyte subpopulations from different zones of cartilage; (2) using a single cell source combined with the correct biochemical and/or biomechanical cues; (3) using different biomaterials and smart scaffold designs. Zonal chondrocyte subpopulations from different zones of cartilage tissue can be harvested [1,166,167], but donor site morbidity, dedifferentiation during expansion, and limited availability are the drawbacks of this strategy. Meanwhile, there is still a debate if zonal chondrocytes can maintain their phenotype after being isolated from their original biomechanical and biochemical environment [31]. Comparing with Strategy (1), Strategy (2) might be an easier and more practical technique using single cell source combined with the suitable biochemical and/or biomechanical cues. BMSCs have been induced to differentiate into zonal chondrogenic cells by co-culturing with various molecules [168,169,170]. This method shows great promise since it would be easier to carry out and potentially could solve the problems associated with direct isolating zonal chondrocytes from cartilage. A good example of Strategy 3 was reported by Wise et al. [171]. They successfully mimicked the cells and ECM organization found in the superficial zone by culturing BMSCs on electrospun and oriented PCL scaffolds. Bio-inks can be formulated based on these strategies for the fabrication of complex zonal structures. Technically, zonal cartilage bioprinting can be realized either by self-supporting hydrogel bioprinting or hybrid bioprinting (Figure 2). It has been reported that zonal engineered cartilage could be fabricated by bioprinting Collagen type II hydrogel constructs with a biomimetic cell density gradient [49]. Even though, zonal cartilage bioprinting is still a challenging task because of the complexity of fabrication process, involving multiple bio-inks preparation, frequent switching between dispensing heads, and complicated real-time calibration.

## 6. Current Limitations and Recommendations for Future Research

EBB is a convenient and promising technique that can print porous tissue-engineered constructs with structural and biological properties from a wide range of bio-inks. It still has several limitations, including limited biomaterials for bio-ink formulation, cell death during printing, low resolution as well as insufficient mechanical properties. Bio-inks formulation is restricted by limited printable biomaterials, which makes up only a small portion of biomaterials applied in TE. To alleviate this problem, development of new biomaterials for bio-ink formulation is needed. When formulating and processing new bio-inks, the properties discussed in Section 3 should be considered and/or compromised for a given CTE application. Further, for clinic application, bio-inks must also satisfy the requirements and regulations as set in standards and norms. Unfortunately, such standards and norms are few nowadays and even none are directly related to bioprinted implants for TE, which raises a great need for such standards and norms [172]. Cell death during the printing process is usually caused by the process-induced forces, such as shear stress, exerted on cells [173,174]. This happens especially when the bio-ink is highly viscous, in which cells would experience significantly higher shear stress [175]. Meanwhile, high viscosity possibly induces clogging of the nozzle tip, leading to disturbance of the printing process [176,177,178]. However, relatively high viscosity is essential for the bio-inks to be dispensed into undisrupted strands with higher resolution and printing accuracy. A recent study printed hydrogels in liquid nitrogen to fabricate scaffolds with high resolution and precisely defined dimensions [179]. However, it impaired the cell viability when printing with cell-laden hydrogels. Therefore, a compromise is usually needed to be made among these factors. Future studies should also focus on new approaches to improve the printability of bio-inks without negatively influencing the cell behavior. Research should also be implemented on developing new techniques to process bio-inks prior to printing to improve printability. For example, increased mixing of alginate and cross-linker solutions actually improved geometric fidelity, mechanical properties, and cell viability of printed constructs [180,181,182].

Other printing parameters, including printing pressure, nozzle geometry and diameter, and bio-ink concentration, have also been shown to influence cell viability within bio-inks [72,130]. Manipulating and optimizing these process parameters can potentially address these issues and challenges to some extent. Recent finding also demonstrated the influence of these printing parameters on printing accuracy [183]. Therefore, we urge that future studies should indicate these parameters when investigating new bio-inks to improve consistency and repeatability.

To fabricate functional cartilage construct, suitable cell sources, biological cues, and construct organization are still needed to be determined for successful cartilage regeneration. Most present studies only focus on evaluating cell viability in different bioprinted hydrogels, while functionality of the engineered cartilage is not very well characterized. As such, we also urge that, for bioprinted engineered cartilage constructs, research should also emphasize the overall chondrogenesis within the constructs, either qualitatively (e.g., Alcian Blue and Safranin O histology) or quantitatively (e.g., collagen and glycosaminoglycan content, or aggrecan and Collagen type II gene expression). Moreover, given that the mechanical performance of cartilage engineered from hydrogel is usually inferior to native cartilage, research on mechanical properties is also required for future cartilage bioprinting studies. Notably, current mechanical characterization of engineered cartilage constructs is mainly performed based on a single mechanical test (Table 4). However, a single acceptable mechanical test result does not sufficiently prove the engineered constructs can perform its biomechanical functions as good as native cartilage tissue. Therefore, a series of mechanical tests (e.g., compression, tensile, and shear tests) needs to be done to comprehensively characterize the mechanical performance of bioprinted cartilage constructs [184].

Theoretically, the shape of scaffolds fabricated by bioprinting techniques can match personalized defects in vivo. Notably, current in vivo research is usually based on man-made regular defects, which can be made fitting with a bioprinted scaffold with exact shape and dimension. It could be difficult for the in vitro printed material to match perfectly with the defect that needs to be regenerated. Printed construct could deform during in vitro culture and defects may expand while waiting for implantation. Although defined defects can be created in clinic, this is not desirable since it further increases the area that needs to be regenerated. Therefore, the concept of “*in situ*” bioprinting has been performed to directly print alginate hydrogels into a defect on an explanted articular surface from a calf [156]. This strategy avoids laboratory-based constructs culture and multiple surgical intervention and would represent the future of TE using bioprinting techniques for cartilage regeneration.

Issues facing CTE is the inability to translate technologies into the clinic and lack of clinic standards of materials for human tissue biopriting [185]. To move bioprinted living cartilage implants into clinic application, bio-inks also must satisfy the requirements and regulations on safety, sterility, and reproducibility. To ensure safety of bioprinted implants for clinical application and to help researchers qualify and validate the bioprinting process and bio-ink formulations, consistent standards are required. Additive manufacturing standards have been published by American Society for Testing and Materials (ASTM) F2792. Meanwhile, standards for tissue-engineered constructs have been approved by the ASTM international committee F04, the International Organization for Standardization technical committee 150/SCZ, and the British Standards Institute. Nevertheless, there are no standards is currently available for bioprinted implants applied in TE field [172]. To ensure sterility throughout bioprinting, the process has to be incorporated in a Good Manufacturing Practice facility, and all components of bioprinter should be sterile and can be operated in a sterile environment. Moreover, the whole bioprinting process should involve minimal manual handling and operation. Therefore, skilled operators are needed to monitor the printing process. Automated, reliable quality control during the printing process will also promote the translation of printers into clinics. Having an integrated bioreactor system with bioprinters to allow in vitro culture before implantation is also an efficient way to avoid undesirable handling of the printed construct and to improve the sterility and reproducibility.

## 7. Conclusions

EBB is an advanced fabrication technique to produce customized cell-laden hydrogel-based constructs for CTE so as to mimic chondral, osteochondral, and zonal organization of articular cartilage. Despite the advantages and opportunities provided by hydrogel-based EBB for cartilage bioprinting, there are still multiple challenges that need to be addressed. Bio-inks for EBB need to be synthesized and optimized in terms of their biocompatibility, formulation, processing, printability, and optimal cell sources. Self-supporting hydrogel bioprinting and hybrid bioprinting are two common approaches to fabricate cartilage constructs. The former technique provides a cell-friendly printing environment but limited mechanical strength, while the latter brings elevated mechanical properties but the stress shielding may disable external mechanical stimuli. Tackling the challenges revolving around bio-inks and mechanical performance of resulting cartilage constructs will foster biologically active and living bioprinted implants for future clinical applications.

## Figures and Tables

**Table 1:** Cell sources that have been used in cartilage tissue engineering (CTE) or cartilage bioprinting.

Cell Source	Features	References for Application in CTE	References for Application in Bioprinting for CTE
**Chondrocytes**			
Artcicular	ease of induction, make it easy to replicate native zonal cartilage by using zonal chondrocytes. Invasive harvesting procedure, donor site morbidity, low cell yields, low bioactivity, tend to dedifferentiate during expansion.	[45,46,47,48,49,50,51]	[48,49,50,51]
Auricular	elastic cartilage, Faster cell proliferation rates than articular chondrocytes, produce more biochemically and histologically similar cartilage than articular chondrocytes when implanted in vivo.	[52,53,54]	–
Nasoseptal	hyaline cartilage, proliferate faster and less tendency of dedifferentiation than articular chondrocytes when culturing monolayer, capable of producing a cartilage ECM with a high GAG accumulation and Collagen type II/I.	[33,34,55,56]	[33,34]
**MSC**			
Bone marrow	high differentiation potentials and less morbidity during harvesting, chondrogenesis under appropriate culture conditions, involving the supplementation of growth factors such as TGF-β, FGF-2.	[38,57,58,59]	[58]
Adipose	differentiating into chondrocytes in the presence of TGF-β, ascorbate, and dexamethasone, lower chondrogenesis. potential than stem cells from other sources, lower deposition of cartilage ECM than other cell types.	[39,60,61]	–
Muscle	differentiation into various lineages, induction to chondrocytes with the addition of BMP-2, improved healing of cartilage defect with an efficacy equivalent to chondrocytes.	[40,41,62,63,64]	–
Synovium	greater chondrogenic potential than stem cells from other sources, comparable biosynthesis level with articular chondrocytes in terms of Collagen type II, aggrecan.	[62,65,66,67]	–
Periosteum	good accessibility, proliferate faster that stem cells from other sources, and capability to differentiate into multiple mesenchymal lineages, including bone and cartilage.	[42,68]	–

**Table 2:** Toolkit of bio-ink formulation.

Materials	Crosslinking	Advantages	Disadvantages	Encapsulated Cells	References in Other Techniques	References in Bioprinting
Agarose	thermal crosslinking at 26–30 °C, extruded agarose solidifies by bioprinting onto a surface of which temperature is lower than the thermal crosslinking temperature	simple and non-toxic crosslinking process, good mechanical properties, and stability of printed construct	not degradable, poor cell adhesion, impaired cell viability due to high temperature to dissolve agarose	bone marrow stem cells(BMSC), adipose stem cells (ASC)	[75,76,77]	[78]
Alginate	ionic crosslinking with divalent cations	rapid gelation, high printability, biocompatible, good stability, and integrality of printed construct	poor cell adhesion, this disadvantage can be overcome by modifying alginate with arginyl glycyl aspartic acid, Collagen type I or oxygenation	BMSC, ASC, chondrocytes	[79,80,81]	[82]
Methylcellulose	thermal crosslinking below 37 °C, silanized hydroxypropyl methylcellulose can be synthesized to be crosslinked by changing pH	good printability, biocompatibility	partially degrade when culturing in cell culture media and therefore not suitable for long-term culturing	chondrocytes	[83,84,85]	[35]
Chitosan	ionic or covalent crosslinking	biocompatibility, antibacterial	slow gelation rate and poor mechanical properties without modification	BMSC	[86,87,88]	[89]
Gellan gum	thermal crosslinking or ionic crosslinking with divalent cation	biocompatible, high printability	poor cellular adhesion	ASC, nasal chondrocytes	[90,91,92]	[93,94]
Hyaluronic acid	ionic or covalent crosslinking, functionalized with methacrylate to be photocrosslinkable	promote cell proliferation, fast gelation, high printability with suitable modification, have lubricating properties	fast degradation, poor mechanical properties and stability without modification	BMSC, chondrocytes, fibroblasts	[95,96,97,98]	[99]
Gelatin	thermal crosslinking, photocrosslinkable polymers can be obtained by functionalization withmethacrylamide side groups to make it stable at 37 °C	biocompatibility, high cell adhesion support cell viability and proliferation	poor mechanical properties and stability, low printability	BMSC, fibroblasts, chondrocytes	[100,101,102]	[69,72,103]
Collagen	pH crosslinking (7–7.4) at 37 °C or thermal crosslinking	biocompatibility, high cell adhesion, promote cell proliferation and serve as a signal transducer, high printability	low gelation rate, poor mechanical properties and stability	BMSC, fibroblasts, chondrocytes	[104,105,106]	[107,108]
Fibrin	enzymatic crosslinking, gels when combining fibrinogen, Ca^2+^ and thrombin at room temperature	biocompatibility, high cell adhesion, rapid gelation	limited printability and poor mechanical properties	BMSC, chondrocytes	[109]	[110,111,112]
Matrigel	irreversible thermal crosslinking at 24–37 °C	biocompatibility, support cell viability and differentiation, high printability	slow gelation and poor stability	BMSC, chondrocytes	[113,114]	[115]
Pluronic^®^ F127	thermal crosslinking	biocompatibility, high printability, support cell viability	weak stability and mechanical properties, fast degradation, slow gelation	BMSC, fibroblasts	[74,116,117]	[118]
Poly(ethylene glycol)	radiation crosslinking or free radical polymerization	biocompatibility, support cell viability, can be easily modified with various functional groups	poor cellular adhesion, low cell proliferation rate	BMSC, chondrocytes	[119,120]	[121]

**Table 3:** Overview of publications on the self-supporting hydrogel bioprinting of (osteo) chondral and zonally organized cartilage regenerative constructs.

Material(s)	Cell Type(s)	Mechanical Properties	Crosslinking Mechanism(s)	Outcomes	Reference
**Hydrogel Bioprinting of Chondral Constructs**					
Alginate	ATDC5 chondrogenic cell line and embryonic chick chondrocytes	Unconfined compressive modulus: 20~70 kPa (depending on the culture time and crosslinking densities)	Ionic	~85% cell viability, show cartilage extracellular matrix formation in constructs	[128]
Nanocellulose with alginate	Human nasoseptal chondrocytes	Unconfined compressive modulus: 75~250 kPa (depending on the ratio of two materials)	Ionic	73–86% cell viability	[34]
Methacrylated chondroitin sulfate (CSMA) with a triblock copolymer poly (N-(2-hydroxypropyl)methacrylamide-mono/dilactate)	ATDC5 chondrogenic cell line	Unconfined compressive modulus: 7–60 kPa (depending on the degree of methacrylation)	Photo	~95% cell viability	[158]
GelMA with gellan gum	ATDC5 chondrogenic cell line	Unconfined compressive modulus: 18–59 kPa (depending on the concentration of gellan gum)	Ionic, photo and thermal	Approximately 50% cell viability in plotted gels due to the supraphysiological temperature of 40–50 °C.	[94,159]
GelMA with gellan gum	Equine articular chondrocytes	Unconfined compressive modulus: 2.7–186 kPa (depending on ratio and content of two components)	Ionic, photo and thermal	Support cartilage matrix production, higher gellan gum contents improves the printability but compromise cartilage ECM, and high total polymer concentrations hamper the distribution of ECM.	[94,159]
Fibroin and gelatin	Human mesenchymal stem cells, Human articular chondrocytes	Not reported	Enzymatic	84–90% cell viability of both cell types during 14 days of culture, supported cartilage ECM deposition and remodeling, minimize hypertrophic differentiation towards development and promote cartilage development.	[73]
Hydroxyethyl methacrylate derivatized dextran (Dex-HEMA) and hyaluronic acid (HA)	Equine articular chondrocytes	Ultimate compressive stress: 100–160 kPa (depending on the HA content), uncontained compressive modulus: 26 kPa for different constructs	Photo	Cell viabilities are 94% and 75% after day 1 and day 3	[153]
Diacrylated Pluronic F127 and methacrylated HA	Bovine articular chondrocytes	Unconfined compressive modulus: 1.5–6.5 kPa (depending on the methacrylated HA content)	Photo	Cell viability is between 60% to 85%.	[152]
GelMA constructs reinforced with methacrylated pHMGCL/PCL	Human articular chondrocytes	Unconfined compressive failure force ~2.7 N and ~7.7 N when covalent bonds between gelMA and methacrylated pHMGCL/PCL are established	Photo	Cartilage ECM network consisting of GAGs and Collagen type II are formed after 6 weeks of in vitro culture and Collagen type II production was more pronounced in vivo compared to in vitro	[29]
Gellan, alginate and cartilage extracellular matrix particles	Bovine articular chondrocytes	Tensile modulus ~116–230 kPa	Ionic and thermal	Cell viability: 80% and 96%, 60% viable cells are observed in the centre of some samples at day 7. Constructs with cartilage ECM particles increased cartilage ECM formation, but the influence of TGF-β3 on cartilage ECM is more pronounced and constructs with TGF-β3 showed most cartilage ECM formation	[27]
Methacrylated HA with HA-pNIPAAM	Bovine articular chondrocytes	Not reported	Thermal and photo	Cell viability is negatively influenced by the addition of HA-pNIPAAM	[28]
**Hydrogel Bioprinting of Osteochondral Constructs**					
Alginate (cartilage) Gelatin with demineralized bone matrix (bone)	Cell-free	Not reported	Ionic	Directly printing into an osteochondral defect of a bovine femur and showed good geometric fidelity	[156]
Alginate (cartilage) Alginate with biphasic calcium phosphate particles (bone)	Human articular chondrocytes (cartilage) Human mesenchymal stromal cells (bone)	Unconfined compressive modulus: 4.5–15 kPa (depending on porosity of constructs)	Ionic	Cell viability: ~89% Cartilage and bone ECM formed in designed regions of the constructs after culturing for 3 weeks. In vivo tests showed similar results after 6 weeks of culture	[157]
GelMA with gellan gum (cartilage) GelMA, gellan gum and polylactic acid microcarriers (bone)	Murine mesenchymal stromal cells	Unconfined compressive modulus: ~25–50 kPa (depending on concentration of microcarriers)	Photo and ionic	Cell viability: 60–90%	[93]
**Hydrogel Bioprinting of Zonally Organized Cartilage Constructs**					
Collagen type II	Rabbit articular chondrocytes (2 × 10^7^ cells/mL in superficial zone, 1 × 10^7^ cell/mL in middle zone and 0.5 × 10^7^ cells/mL in deep zone)	Not reported	Thermal	Cell viability: 93% Zonally organized cartilage constructs could be fabricated by bioprinting Collagen type II hydrogel constructs with a biomimetic cell density gradient. The cell density gradient distribution resulted in a gradient distribution of ECM	[49]

**Table 4:** Overview of publications on the hybrid bioprinting of osteo (chondral) constructs.

Material(s)	Cell Type(s)	Mechanical Properties	Crosslinking Mechanism(s)	Outcomes	Reference
**Hybrid Bioprinting of Chondral Constructs**					
Alginate reinforced with polycaprolactone (PCL) framework	C20A4 human chondrocyte cell line	Unconfined compressive modulus: 6000 kPa	Ionic	Cell viability varies from 70 to 80%. Co-deposition of thermoplastic polymer and hydrogel is firstly introduced for bioprinting of reinforced constructs.	[141]
Alginate reinforced with PCL framework	Human nasoseptal chondrocytes	Not reported	Ionic	85% cell viability, cartilage ECM formation in constructs with the addition of TGF-β after culturing for 4 weeks. Cartilage ECM formation is observed in constructs with after 4 weeks in vivo.	[33]
Alginate reinforced with PCL framework	Embryonic chick chondrocytes	Not reported	Ionic	Cell viability: 77–85%; Cartilage ECM (glycosaminoglycan and Collagen type II) is formed in constructs.	[160]
Decellularized extracellular matrix (dECM) reinforced with PCL framework	Human adipose-derived stem cells (hASCs) and human inferior turbinate-tissue derived mesenchymal stromal cells (hTMSCs)	Not reported	Thermal	Cell viability: >90%. The dECM provided cues for cells survival and long-term functionality. Embedded cell synthesizes cartilage ECM and expressed chondrogenic genes.	[143]
**Hybrid Bioprinting of Osteochondral Constructs**					
Alginate reinforced with PCL framework	Human nasoseptal chondrocytes (cartilage) Human osteoblasts cell line (MG63)	Not reported	Ionic	Cell viability: ~93.9% for dispensed chondrocytes and ~95.6% for dispensed osteoblasts during 7 days of culture.	[161]
Atelocollagen supplemented with BMP-2 (cartilage) CB[6]-HA supplemented with TGF-β (bone) The whole structure is reinforced with PCL framework	Human turbinate-derived mesenchymal stromal cells (hTMSCs)	Not reported	Thermal and enzymic	Cell viability: 93% for atelocollagen (bone) and 86% CB (6)-HA (cartilage). In vivo results showed neocartilage is formed in cartilage region while new bone is observed in subchondral bone. The constructs are well integrated with surrounding native tissue in vivo.	[162]
